# Application of Virtual Reality to Alter Sweetness Perception †

**DOI:** 10.3390/foods15122150

**Published:** 2026-06-14

**Authors:** Serena Wellbelove, John Gieng, Valerie Carr, Kate McLeod, Xi Feng

**Affiliations:** 1 Department of Nutrition, Food Science, and Packaging, San José State University, San José, CA 95192, USA; serenastarr3@gmail.com (S.W.); john.gieng@sjsu.edu (J.G.); mary.mcleod@sjsu.edu (K.M.); 2 Department of Psychology, San José State University, San José, CA 95192, USA; valerie.carr@sjsu.edu

**Keywords:** virtual reality, sugar-sweetened beverages, sweet perception, sensory analysis

## Abstract

Regular consumption of excess sugar is linked to nutrition-based diseases, including gut problems, Non-Alcoholic Fatty Liver Disease, and Type 2 Diabetes Mellitus. Increasing sweetness perception is a novel technique to decrease sugar consumption. This experiment compared the sweetness perception of sweetened and unsweetened almond milk in response to different virtual environments with music and visuals. Two music types, the classical song Goldberg Variations, BMV. 998-Variation 13 and a jazz song generated by AI were used. Additionally, fall and spring forest backgrounds were generated by the Blockade Labs 3D image generator. Each participant tasted sweetened and unsweetened almond milk in music-only, background-only, and combination music and background environments. Results revealed significant differences in sweetness ratings for music type (*p* = 0.015) and between milk types (*p* < 0.001). Viscosity rating differed significantly between backgrounds (*p* = 0.04) and by milk type (*p* < 0.001). Liking ratings varied significantly between backgrounds (*p* < 0.001) and between music genres (*p* = 0.011). The results suggest that altering music and background may be a strategy to change sweetness and viscosity perception in unsweetened beverages.

## 1. Introduction

According to the CDC 2026 National Diabetes Statistics Report, 40.1 million Americans have diabetes, approximately 12% of the population and 40% of U.S. adults are obese [1]. Poor dietary habits, physical inactivity, genetic reasons, etc., are the factors that contribute to diabetes and obesity [2]. Overconsumption of sugar is a common problem in Western diets. Current guidelines suggest limiting added sugar to 10% or less of total calorie intake per day, yet the average American diet includes 13% of calories from sugar. Eating high-sugar foods may lead to a reduction in cortisol levels, with highly stressed individuals choosing higher-fat and sugar foods [3,4,5]. Consuming large amounts of high fructose corn syrup may disrupt the gut microbiome [6]. The human gut microbiome changes based on diet, a process that starts to happen as young as six months of age. For example, Mokhtari et al. (2024) found that infants who consume high levels of added sugars display less abundance in gut microbiota than infants whose diets are high in insoluble fiber [7].

Western diets are thought to increase instances of microbiota flora associated with non-alcoholic fatty liver disease [8]. Additionally, increased intake of sugar-sweetened beverages leads to a 16% higher chance of developing type two diabetes mellitus, while diets high in ultra-processed foods may increase risk by 53% when compared to recommended consumption [9,10]. Furthermore, regular consumption of soft drinks leads to a greater risk of developing metabolic syndrome than solid sugar consumption [11]. High sugar diets are linked to visceral adiposity and hypertriglyceridemia as well as higher body weight in rats [12].

Strategies to lower sugar consumption include taxation, sugar replacement, and artificial sweeteners. Sugar taxes are used to disincentivize the purchase of sugar-sweetened beverages [13]. Reducing sugar content in products to decrease overall intake uses five main tactics: reducing overall sugar, replacing the sugar, removing the sugar, boosting the sweetness to make small amounts of sweetener taste like more, and blocking sweetness receptors or absorption. One of the most common methods used to reduce sugar consumption is the addition of artificial sweeteners. While artificial sweeteners may reduce weight in the short term, the correlation between long-term use and weight maintenance is not clear, as no long-term weight maintenance benefits are seen [14]. Additionally, simply switching from sugar-sweetened to artificially sweetened beverages does not lead to a lower risk for type two diabetes mellitus and may increase the risk for other metabolic disorders [15]. Aspartame and acesulfame-K, two commonly used artificial sweeteners, are linked with increased risk for breast and obesity-related cancers [16]. Furthermore, not all artificial sweeteners are thermally stable and are unable to simply be exchanged for glucose in many products. Sugar-sweetened beverages are a common source of added sugar [17]. One increasingly common sweetened beverage is plant-based milk. Studies indicate sales of plant-based milks increased rapidly from 2004 to 2018, with demand expected to continue rising [18,19]. Increasing sales may be due to the consumer association that plant-based milks are healthy and sustainable [20]. Almond milk is the largest segment of the plant-based milk category, which accounts for 49% in 2025 [21].

Sensory environments can alter the experience of eating food, and factors such as background noise and color are novel targets to manipulate food consumer opinions. Noise sensitivity, loud noises, and music type have all been shown to alter food perception [22,23,24,25,26]. A study performed by Guedes et al. (2023) found that participants listening to music played at 80 beats per minute had an increased preference for sweet snacks [24]. Similarly, in research by Motoki et al. (2022), participants who listened to Bach’s Goldenberg Variations selected more sweet products over savory than participants listening to jazz [27]. Additionally, red color tends to be associated with sweet flavors, while green colors are seen as an incongruent match to sweetness [28]. Goel and Grasso found that young men and women had a higher odor-discrimination ability in the fall season than in winter or spring [29]. Lemos et al. (2020) found that participants shown a red code had positive feelings towards ultra-processed foods despite the lower nutritional value [28]. However, Cornelio et al. (2022) found that light alone is not immersive enough to alter the experience of sweetness, indicating added environment may be necessary to change sweetness perception [30]. Potentially, altering an environment to include music and an immersive background, such as virtual reality, may increase sweetness perception. Currently, virtual reality has been used in nutrition interventions and education [31,32].

The viscosity of liquids may impact their flavor and palatability. A study on rats found that viscosity preference was able to be appetitively conditioned using a xanthium gum thickened solution with added nutrients [33]. After conditioning, rats showed a greater preference for viscous liquids, indicating that viscosity can contribute to taste preference. Highly viscous liquids may have overall lower palatability than low-viscosity liquids [34]. However, when a viscous liquid has a favorable flavor, viscosity perception is reduced, indicating that individuals notice viscosity less when they are drinking something they enjoy. Furthermore, individual texture preferences impact perception as much as exposure and interest in the food [35]. Research by Kamei et al. (2024) found that smoothness perception was impacted by preference for smooth texture [35]. A greater individual preference for smooth textures was positively associated with the amount of smoothness perceived. A person eating food that matches their preferred texture will have higher enjoyment and higher texture perception than someone eating food that does not match their texture preferences.

The following study used VR to manipulate the environment and sound in sensory evaluation tests of sweetness perception and viscosity. The project aimed to deepen the knowledge of these sensory characteristics through controlled evaluation of products in novel circumstances as a potential method to decrease sugar consumption [36]. This functioned as targeted market research that may be applied in future product development [36]. Additionally, the project introduced environmental context into the sensory evaluation experience, helping to further develop the understanding of how music and environment can influence sweetness perception [37]. Almond milk provided an easy-to-test sugar-sweetened beverage in this experiment because it can be purchased in both sweetened and unsweetened versions, allowing added sugar to be clearly evaluated. This product was appropriate for this test because it was easy for participants to consume in VR with a lid and a straw, which is easy to drink from and hard to spill.

Our study manipulated three variables: music (classical, jazz, or none), background (spring, fall, or none), and almond milk type (sweetened, unsweetened). With respect to music, we hypothesized that both classical and jazz music would lead to higher sweetness ratings than no music [24,28]. For background, we predicted that a red fall forest background would increase sweetness perception relative to a green spring background or no background, as seen in previous research by Lemos et al. (2020) [28]. Finally, we hypothesized that combining either music type with the red fall forest background would lead to higher sweetness perception than music alone or the red fall background alone, as well as higher sweetness perception than a combination of either music type with the green forest background. We expected each of these hypotheses to hold true for both sweetened and unsweetened almond milk, with larger effects seen for unsweetened almond milk. There is no research about viscosity with music types and background environments; we would like to test in this study, as it is an important sensory characteristic in almond milk [38].

## 2. Materials and Methods

### 2.1. Participants

Participants were selected from San José State University (San José, CA 95192, USA). No specific demographics, such as age, gender, or ethnic background, were targeted. Inclusion criteria were an affiliation with San Jose State, being 18 years or older, and being free from allergies to any ingredients in the almond milks. Marketing materials in the form of physical and digital flyers were posted around the San José State University campus and emailed to students. A priori power analysis was conducted using Cohen’s conventional medium effect size assumption (*f* = 0.25). No pilot data were available to estimate an expected effect size for this VR-based almond milk sensory-evaluation study; therefore, *f* = 0.25 was used as a conventional planning assumption. The analysis indicated that 48 participants were required to achieve 80% power. Recruitment continued until this target was exceeded, resulting in a final sample of 54 participants. Participants were offered a $10 Amazon gift card as a reimbursement for their time. No demographic data was collected as the research was not targeting a specific demographic.

### 2.2. Experimental Design

**Figure 1:**
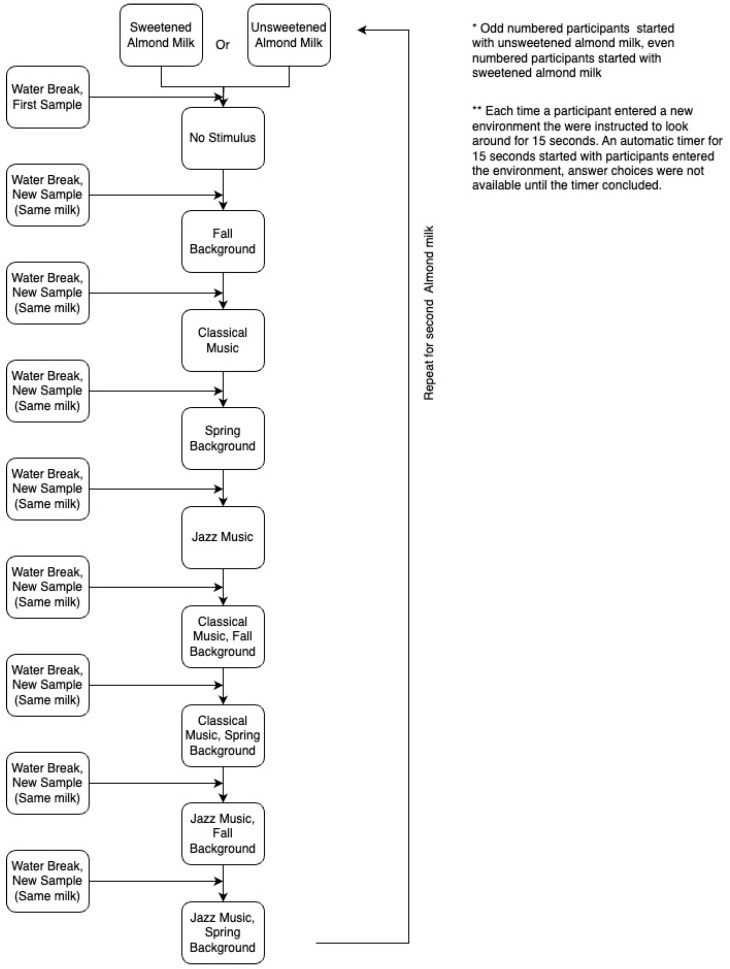
Flow chart of sensory evaluation test.

The research performed was a quasi-experimental study. All the participants evaluated 2 almond milks in 9 different environments (no stimulus, fall and spring seasons, classical and jazz music, and their combinations). The independent variables were environment, milk type, and music. The dependent variable was the participant’s rating of sweetness perception and viscosity of sweetened and unsweetened almond milk and liking of the music and environments. In the no-stimulus environment, participants only evaluated sweetness and viscosity. The liking of the environment was not rated. In the stimulus environment, participants evaluated their liking of the environment, the sweetness and the viscosity of the almond milk. Participants were presented with a 9-point Likert scale in a virtual reality headset where they completed 52 sensory evaluation questions as shown in Figure 1. Silk brand unsweetened, nutrition facts (per 240 mL serving): fat 3 g; total carbohydrate 1 g; protein 1 g, and sweetened almond milk, nutrition facts (per 240 mL serving): fat 2.5 g; total carbohydrate 8 g (including 7 g added sugars); protein 1 g, were purchased from local supermarkets and then stored at 4 °C for the experiment.

### 2.3. VR Android Package Kit

**Figure 2:**
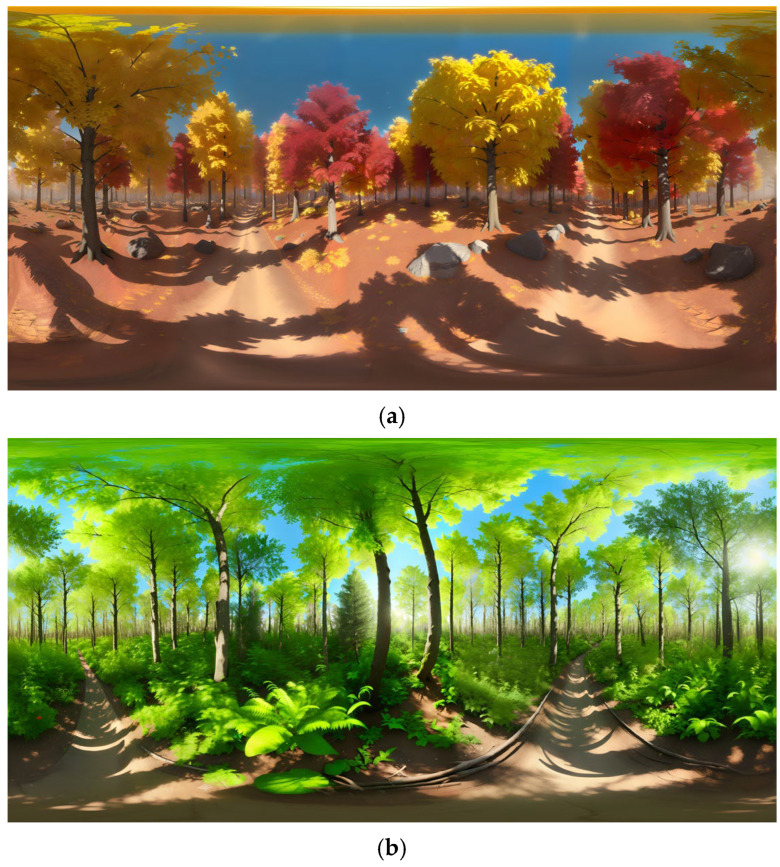
360-degree virtual reality (VR) backgrounds created with AI: (**a**) fall forest and (**b**) spring forest.

A bespoke VR Android package kit (APK) was created on meta-Oculus to run the experiment. Nine different virtual backgrounds were created: a control environment (a gray background without music), music-only environments (classical or jazz), background-only environments (fall or spring), and combination background and music environments (classical music and fall background, classical music and spring background, jazz music and fall background, and jazz music and spring background) (Figure 1). Participants each went through the experiment in the same order due to practical limitations in the VR coding and experimental resources. The classical song used was the Goldberg Variations, BMV. 998-Variation 13, which was chosen based on the finding that this song increased preference for sweet foods by Motoki et al. (2022) [27]. The jazz song was generated by Music FX using the prompt, “jazz song, a call in the bass followed by a response from the piano and horns the response part is a pair of minor seventh chords a whole step apart” and was based on the findings of Guedes et al. (2023) that 80 BPM music increased preference for sweet foods [24,39]. The two forest backgrounds were created using Blockade Labs 3D image generator [40]. One forest was predominantly green and known as the spring environment while the other was predominantly red and known as the fall environment, motivated by the findings by Lemos et al. (2020) that red color increases preference for sweet foods while green color does not [28]. The fall environment was based on the prompt, “autumn forest, everything red and orange filling you with a sense of fall (Figure 2).” The spring environment was created with the prompt, “spring forest, everything is green and bursting with life (Figure 2).” Once the APK was completed, the program was loaded onto meta-Oculus devices (Meta Platforms, Inc. Menlo Park, CA, United States).

### 2.4. Sensory Evaluation

**Figure 3:**
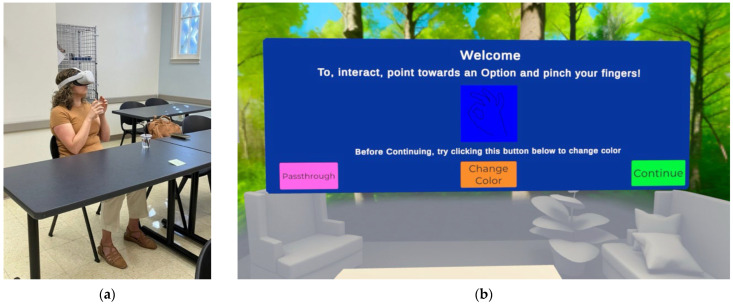
(**a**) Participant in sensory evaluation with meta-Oculus; (**b**) participant’s view of Oculus welcome screen.

The experiment took place in the San José State University Central Classroom Building. Participants were seated at a table with one Oculus headset set to hand tracking mode, a consent form, and an ID number (Figure 3). The Oculus headset was turned on, and the APK app created for the experiment was opened. The Oculus had hand tracking enabled, allowing participants to use their hands to select options during the experiment. The researcher reviewed how to use the Oculus and what to expect in the experiment before the participant began. Participants were told they were able to immediately end the experiment if they no longer wished to participate, experienced any motion sickness, experienced adverse effects in the virtual reality, or experienced adverse effects from the almond milk. Each participant placed the Oculus on their head and adjusted the headset to fit comfortably with the help of the researcher. In the Oculus, participants could see a welcome environment of gray chairs and a welcome screen with a hand in a colored background and three buttons reading pass through, change color, and continue (Figure 3). The “passthrough button” was to enable researchers to see the APK app at the same time as the Oculus menu. The “change color button” changed the background color of the hand and was included for participants to practice making selections on the Oculus. Participants were told to practice changing the color of the background of the hand until they felt comfortable using their hand to control the Oculus. The “continue button” started the experiment. Participants then selected continue and were prompted to enter their randomly assigned ID number. Once the ID number was entered, participants were able to begin the experiment.

**Figure 4:**
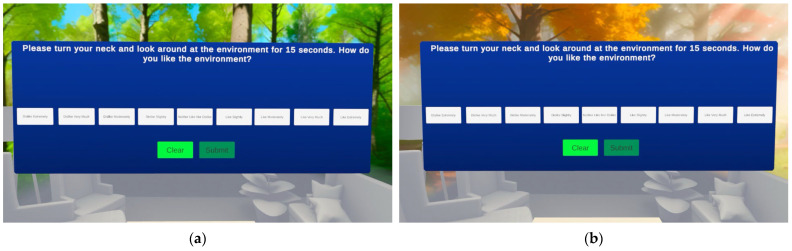
Participant view of sample questions in (**a**) spring environment and (**b**) fall environment.

Once participants began the experiment, the water cup and the first sample were placed on the table. Participants started the experiment with a water break to rinse out their mouths. The order of environments and water breaks is outlined in Figure 1. Participants received one sample (30 mL) at a time to evaluate. Participants followed the guidance provided in each question to evaluate the following sensory characteristics: sweetness intensity, viscosity intensity, and liking of environment on a 9-point Likert scale in a virtual reality headset (Figure 4). The term “viscosity” was defined in each relevant sensory evaluation prompt and participants were provided instructions for how to drink the beverage so that the intended characteristic was correctly evaluated on the nine-point Likert scale. Responses were self-paced. Each answer was recorded in real-time on a secure server monitored by the researcher. As participants completed each milk sample the researcher brought the next sample while the participant completed their water breaks. Participants were free to leave after completing all the samples.

### 2.5. Statistical Analysis

Data was analyzed using SPSS software version 29.0.2.0 by IBM. A mixed linear model test (MLM) was employed. The dependent variables tested were sweetness rating (intensity), viscosity rating (intensity) and liking (environment). The measured fixed effects were milk type (sweetened vs. unsweetened), background (fall, spring, none), and music (classical, jazz, none). The full factorial model included the main effects of background, music, and milk type, all two-way interactions (background × music, background × milk type, and music × milk type), and the three-way interaction (background × music × milk type). Thus, the primary model was: outcome = background + music + milk type + background × music + background × milk type + music × milk type + background × music × milk type + participant random intercept + repeated trial effect. A random intercept for subjects was used to account for within-subject correlations. A diagonal covariance structure was used to model repeated measures. Parameter estimation was conducted using Restricted Maximum Likelihood (REML). Pairwise comparisons were adjusted using the Bonferroni correction. Additionally, univariate analysis tests were run to test the effects of background and music on both sweetness rating and viscosity rating by milk type (sweetened or unsweetened). Approximate partial eta-squared values (ηp^2^) were calculated from the fixed-effect F tests to aid interpretation of effect magnitude. To evaluate potential presentation-order bias, sensitivity analyses were conducted using stimulus order as an order-related covariate. Because VR/music environments were presented in a fixed order, this analysis assessed whether ratings changed systematically across the session but could not fully separate order effects from stimulus effects.

## 3. Results

### 3.1. Impact of Environment on Sweetness Rating

No significant two-way or three-way interactions affected sweetness perception. The average sweetness rating was significantly impacted by the music-only environments (*p* = 0.015) and milk type (*p* < 0.001) (Table 1). The perception of sweetness increased in VR settings where participants were only listening to music and were not shown a background. Sweetened almond milk was rated as significantly sweeter than unsweetened almond milk. Effect-size estimates indicated that milk type had the strongest effect on sweetness rating (ηp^2^ = 0.418), whereas the effect of music was small (ηp^2^ = 0.018). The type of VR environment had negligible effects compared to milk type which demonstrates the difference in perceived sweetness resulting from added sugar. The different ratings between VR environments demonstrate the potential range of perception manipulation compared to the effect of physically changing a consumer product, outlining the commercial limitations of applying this concept in product development (Table 2 and Table 3). Participant ratings revealed the predominant color of the forest background had no significant impact on sweetness perception (*p* = 0.304) (Table 1).

The combination of both music and background effects did not significantly impact sweetness perception overall. The combination environment was significantly associated with sweetness perception in unsweetened milk (*p* = 0.02) (Table 4). However, Bonferroni-adjusted pairwise comparisons did not identify specific combined music/background conditions that differed significantly. These results suggest that the cross-modal interactions between actual sugar content, music, environment, and sensory attributes are dynamic and interrelated in their impact on consumer preference.

### 3.2. Impact of Environment on Viscosity Rating

No significant two-way or three-way interactions affected participant perception of viscosity. In contrast to sweetness perception, which was impacted by music, the average viscosity rating was significantly impacted by the background-only environments (*p* = 0.04) and milk type (*p* < 0.001) (Table 1). Sweetened almond milk was rated as significantly more viscous than unsweetened almond milk. Effect-size estimates indicated small effects of background (ηp^2^ = 0.012) and milk type (ηp^2^ = 0.038) on viscosity rating. Despite this, viscosity was not significantly affected by the combination of music and VR environments for either sweetened or unsweetened milk (Table 4).

### 3.3. Impacts of Environment on Liking of Music and Background

**Figure 5:**
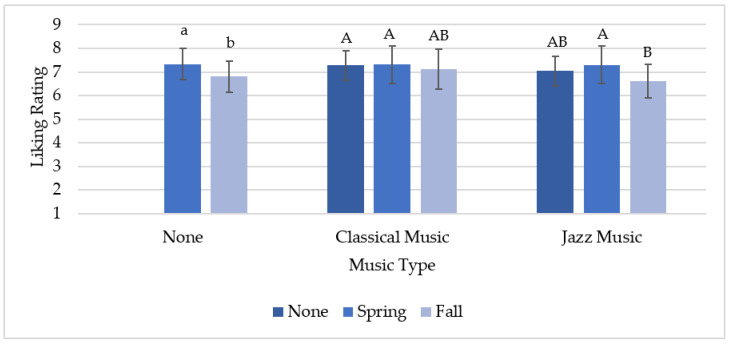
Participant liking rating of environments (mean and standard deviation) in different music and virtual environment combinations. Different uppercase letters indicate significant differences among music/background combination conditions based on Bonferroni-adjusted post hoc comparisons. Different lowercase letters indicate significant differences between background-only conditions.

No significant two-way or three-way interactions were seen to alter the liking of music and background. The preference for music and background was significantly different in background-only environments (*p* < 0.001) and music-only environments (*p* = 0.011) (Table 1 and Figure 5). Effect-size estimates indicated a modest effect of background (ηp^2^ = 0.068) and a small effect of music (ηp^2^ = 0.012) on liking rating. This result in background-only and music-only environments may suggest individual music and background preferences or may demonstrate the confounding effects of receiving both visual and auditory stimuli. The preference for music and background was not related to the type of milk the participant was drinking or the actual or perceived sweetness of that beverage.

Sensitivity analyses showed no significant presentation-order effect for sweetness or viscosity ratings. However, stimulus order was significantly associated with liking ratings (*p* = 0.011, ηp^2^ = 0.049), suggesting that liking ratings changed slightly across the presentation sequence.

## 4. Discussion

This study aimed to use virtual reality to manipulate the consumer environment and impact sweetness perception and viscosity of almond milk. The study investigated the impacts of different background environments and musical environments, both separately and combined, to measure differences in perception.

Sweetness perception was significantly altered by music type and by milk type, supporting the hypothesis that music would increase sweetness perception. The impact of music on sweetness is consistent with the findings of Campinho et al. (2023) that auditory stimuli influence taste perception [41]. The significance in sweetness ratings of almond milk with music alone furthers the research into this topic, yet more investigation is needed into the potential applications of these musical and sensory phenomena. In this study the fall environment was chosen to have predominantly red colors which have been associated with sweet flavors while the spring forest was designed to have predominantly green colors which are not associated with sweetness [42]. These previous color associations were not supported by our results, as the VR visual environment was not associated with participants’ sweetness ratings. The forest backgrounds that were used in this study may not have been contextual to drinking almond milk so this lack of standard context may have impacted these findings. This is further supported by a study testing wine under green or red lighting. In the red lighting with sweet music, participants had a higher likeness [43].

Milk type had the highest effect on sweetness ratings, with sweetened almond milk (sugar content: 2.9 g/mL) being significantly sweeter than unsweetened almond milk (sugar content: 0 g/mL). The high impact of milk type mirrored the findings of Torrico et al. (2021) who similarly found that fully sweetened chocolate had higher sweetness intensity than chocolates with no-sugar maltitol [44]. These results demonstrate that physical changes (such as adding more sugar) are the most direct way to change sweetness, despite the potential effects of manipulating the external environment. This was consistent with our finding of no changes in sweetness perception in any of the combination environments except for unsweetened milk. Research by Mathiesen et al. (2022) found that the interactions between multisensory elements were imperative in predicting desire for comfort food, demonstrating the complexity of altering the combined total effect of multiple sensory inputs [45]. Our results highlighted these complex interactions between milk type, music, and visual environment. Viscosity rating of almond milks varied significantly in background-only environments which implied that external visual stimuli may impact viscosity perception. Viscosity rating was also significantly different by milk type, further demonstrating that milk type has the strongest influence on sensory attributes. Effect-size estimates further indicated that milk type had the strongest effect on sweetness rating, whereas the effects of music on sweetness, background on viscosity, and music/background context on liking were smaller and should be interpreted cautiously. More research is needed to fully understand the interrelated impacts of music and background on sensory ratings.

## 5. Limitations

These findings suggest that music may modestly influence perceived sweetness under controlled VR testing conditions. The primary limitation of this study is the lack of liking rating data in the combined no background and no music environment. Due to the lack of this data point, the other liking ratings could not be compared to a control value. Participant demographic information was not collected in this study so no correlations could be made on this topic. The experiment used red and green forest backgrounds, but approximately 8% of the male population is red/green color blind [46]. Color blindness was not an exclusion criterion in the experiment yet results from colorblind participants may have impacted overall findings. Although the milk type order was randomized, the VR/music environments were presented in a fixed order because of VR programming and resource constraints. Sensitivity analyses did not indicate significant order effects for sweetness or viscosity, but stimulus order was significantly associated with liking ratings. Because the stimulus order was fixed, the significant order effect for liking cannot be fully separated from the specific VR/music conditions presented later in the sequence. Repeated evaluations may also have introduced order bias, sensory fatigue, novelty effects, or demand characteristics. Future studies should randomize or counterbalance the presentation order of VR/music stimuli to better separate stimulus effects from presentation-order effects. Although serving temperature may impact overall taste perception, prior research indicates temperature does not have significant effects on sweetness perception and should not be considered a limitation in these findings [47]. Hunger level, stress levels, music tastes, and liking of almond milk were not collected as part of the data in the experiment. As only two samples were tested in eight different environments in this study, more studies should be conducted in different samples and populations.

## 6. Conclusions

The results of this sensory study provide insight into how sweetness perception and viscosity may be influenced by the external environment. Sweetness perception of almond milk was altered in music-only environments, demonstrating that the setting can be manipulated to influence sweetness perception. These results could be used by product developers to help identify factors that influence product acceptability and consumer behavior. By using VR environments product developers could quickly and cost-effectively gain a better understanding of consumer acceptance. As VR is not easily accessible due to costs, and consuming foods in VR can be challenging, future research should focus on finding more proof-of-concept evidence and how these findings may be extrapolated to real-world settings. Related studies may focus on other product types or different sensory attributes such as salt perception. The masking effect of added sugar, novelty of environment, emotional state, and time of day are also related study targets in sensory analysis. With increased knowledge, food developers will have a greater understanding of the impact of the environment on sweetness perception, and this knowledge may allow businesses to selectively manipulate the sweetness of their products. Ultimately, this body of research may reveal specific environmental factors that could be used to increase sweetness perception [48].

## Figures and Tables

**Table 1:** Sensory ratings by main model factor.

Background	Music	Milk Type	Sweetness Rating (Mean ± SD)	*p*-Value; ηp^2^	Viscosity Rating (Mean ± SD)	*p*-Value; ηp^2^	Liking Rating (Mean ± SD)	*p*-Value; ηp^2^
Fall			5.7 ± 1.1	0.304; 0.005	4.4 ± 1.7	0.04; 0.012	6.9 ± 1.3	<0.001; 0.068
Spring			5.7 ± 1.1		4.6 ± 1.7		7.3 ± 1.3	
None			5.1 ± 1.2		4.3 ± 1.7			
	Classical		5.7 ± 1.1	0.015; 0.018	4.5 ± 1.7	0.096; 0.009	7.3 ± 1.2	0.011; 0.012
	Jazz		5.8 ± 1.1		4.4 ± 1.7		7.0 ± 1.2	
	None		5.4 ± 1.2		4.3 ± 1.7			
		Unsweetened	4.5 ± 1.1	<0.001; 0.418	4.2 ± 1.7	<0.001; 0.038		
		Sweetened	6.8 ± 1.0		4.7 ± 1.7			
Background × Music				0.654		0.074		0.085
Background × Milk Type				0.302		0.454		
Music × Milk Type				0.124		0.478		
Background × Music × Milk Type				0.219		0.779		

Note: Values are reported as mean ± SD. *p*-values and approximate partial eta-squared values (ηp^2^) are from mixed-effects models including background, music, milk type, and their interactions as fixed effects, participant as a random intercept, and trial number as a repeated effect. Liking was analyzed only for conditions in which background and/or music liking was collected.

**Table 2:** Sensory ratings for unsweetened almond milk.

Background	Music	Sweetness Rating (Mean ± SD)	*p*-Value	Viscosity Rating (Mean ± SD)	*p*-Value	Liking Rating (Mean ± SD)	*p*-Value
None	Classical	4.6 ± 1.6	<0.001	4.4 ± 1.7	0.042	7.3 ± 1.2	0.085
	Jazz	4.7 ± 1.6		4.2 ± 1.7		7.0 ± 1.2	
	None	4.2 ± 1.7		4.1 ± 1.7			
Fall	None	4.5 ± 1.6	0.046	4.1 ± 1.7	0.05	6.8 ± 1.3	0.014
Spring		4.6 ± 1.6		4.4 ± 1.7		7.3 ± 1.3	
None		4.3 ± 1.7		4.1 ± 1.7			
Fall	Classical	4.7 ± 1.7	0.116	4.3 ± 1.9	0.051	7.1 ± 1.7	0.342
	Jazz	5.0 ± 1.8		4.2 ± 1.9		6.6 ± 1.4	
Spring	Classical	4.9 ± 1.9		4.6 ± 1.9		7.3 ±1.6	
	Jazz	4.8 ± 1.9		4.1 ± 1.9		7.3 ± 1.6	

Note: Values are reported as mean ± SD. *p*-values are from mixed-model or simple-effect tests for the listed background, music, or combined background/music condition. Liking ratings were available only for conditions in which background and/or music liking was collected.

**Table 3:** Sensory ratings for sweetened almond milk.

Background	Music	Sweetness Rating (Mean ± SD)	*p*-Value	Viscosity Rating (Mean ± SD)	*p*-Value	Liking Rating (Mean ± SD)	*p*-Value
None	Classical	6.8 ± 0.9	0.42	4.7 ± 1.2	0.085	7.3 ± 0.9	0.188
	Jazz	6.8 ± 0.9		4.7 ± 1.2		7.0 ± 0.1	
	None	6.7 ± 1.0		4.6 ± 1.2			
Fall	None	6.8 ± 0.9	0.147	4.7 ± 1.2	0.456	6.9 ± 1.2	0.087
Spring		6.7 ± 0.9		4.7 ± 1.2		7.3 ± 1.2	
None		6.7 ± 1.0		4.5 ± 1.2			
Fall	Classical	6.9 ± 1.6	0.622	4.7 ± 2.1	0.144	7.1 ± 1.7	0.537
	Jazz	6.7 ± 1.5		4.8 ± 2.1		6.6 ± 1.7	
Spring	Classical	6.9 ± 1.6		4.8 ± 2.1		7.4 ± 1.7	
	Jazz	6.6 ± 1.7		4.6 ± 2.0		7.3 ± 1.8	

Note: Values are reported as mean ± SD. *p*-values are from mixed-model or simple-effect tests for the listed background, music, or combined background/music condition. Liking ratings were available only for conditions in which background and/or music liking was collected.

**Table 4:** Simple effects of combined background/music condition within each milk type.

Milk Type	Sweetness *p*-Value	Sweetness ηp^2^	Viscosity *p*-Value	Viscosity ηp^2^
Unsweetened Milk	0.02	0.187	0.061	0.146
Sweetened Milk	0.727	0.065	0.748	0.059

Note: Values are from the mixed-effects model including combined background/music condition, milk type, and their interaction as fixed effects. Participant was modeled as a random intercept, and trial number was modeled as a repeated effect. Effect sizes are reported as approximate partial eta squared, ηp^2^.

## Data Availability

The original contributions presented in this study are included in the article. Further inquiries can be directed to the corresponding author.
